# Does the implicit models of leadership influence the scanning of other-race faces in adults?

**DOI:** 10.1371/journal.pone.0179058

**Published:** 2017-07-07

**Authors:** Iain L. Densten, Luc Borrowman

**Affiliations:** 1 Monash University Australia Alumni, Melbourne, Australia; 2 Department of Economics, School of Business, Monash University Malaysia, Sunway, Malaysia; Leiden University, NETHERLANDS

## Abstract

The current study aims to identify the relationships between implicit leadership theoretical (ILT) prototypes / anti-prototype and five facial features (i.e., nasion, upper nose, lower nose, and upper lip) of a leader from a different race than respondents. A sample of 81 Asian respondents viewed a 30-second video of a Caucasian female who in a non-engaging manner talked about her career achievements. As participants watch the video, their eye movements were recorded via an eye tracking devise. While previous research has identified that ILT influences perceptional and attitudinal ratings of leaders, the current study extends these findings by confirming the impact of ILT on the gaze patterns of other race participants, who appear to adopt system one type thinking. This study advances our understanding in how cognitive categories or schemas influence the physicality of individuals (i.e., eye gaze or movements). Finally, this study confirms that individual ILT factors have a relationship with the eye movements of participants and suggests future research directions.

## Introduction

Traditionally, leadership research has focused on the leader and their display of traits, values, and behaviors. Such a focus has resulted in the role of followers and the context being under-represented in the literature [1]. As a result, our understanding of follower judgments and their comparisons about their leaders is less than adequate. Moving to a more follower-centered focus enables the investigation of raters’ subjective views of leaders and followers to be better understood [2, 3]. Such a follower-centered approach could have a profound impact on the leader/leadership development, which employs numerous questionnaires to gain insights about executive and non-executive leaders. Currently, the Implicit Leadership Theories (ILT) are being used to fill in this neglected part of the leadership literature by uncovering the implicit benchmarks or prototypes of leaders and followers that are activated in different contextual experiences. The current study uses a video to investigate the prototypes and anti-prototypes of followers by assessing their eye movement reaction to a video presenting a reputed leader.

Understanding these prototypes used by followers can provide critical information about how they both understand and respond to managerial behaviors. After all, previous research has discovered that individuals can distinguish leaders from non-leaders according to features [4, 5]. Lord and his colleagues suggest that such features or prototypes reside in our long-term memory and consists of a large and well-elaborated belief system [4, 5] and even enables the features that distinguish leaders from non-leaders to be more clearly identified. These leader prototypes and anti-prototypes are multidimensional, widely shared, trait-based knowledge or memory structures that are formed very early in life [4–6]. Such prototypes focus on the underlying cognitive pattern–matching processes that are used to organize memories of leadership and guide the processing of leadership information that mediates our leadership perceptions [3, 7]. These leadership perspectives occur during social interactions that involve a range of senses which include the eyes of individuals.

### Role of the follower rater

Typically, the role of follower raters has been limited to providing retrospective frequency judgments from items in questionnaires (e.g., how often does your leader use emotive language) that are reported to reflect underlying leader behaviors [8]. Such judgments trigger the use of prototypes [7], which are based on patterns of information aggregated across many events [9, 10] Reliance on these patterns (i.e., schemas, scripts, and social categories) may introduce bias into behavioral ratings of leadership [8]. False memories can be produced through reliance on prototypical leader behavior when feelings of familiarity are heightened [11].

ILT has always offered the potential to gain important insights into the process of sense-making within organizations [12]. However, recognition of the importance of ILT has been impeded by early studies viewing it as a source of bias in leadership measurement [e.g., 2, 13] and discounting its key role in the interpreting and generation of behaviors [14]. Research by Lord et al., [3] and Cronshaw and Lord [15] have helped establish our understanding of how ILTs effect the perception of leadership and its capacity as an explanatory framework for organizational leadership. Such work builds upon the Theory of Cognitive Categorization by Rosch [16], which according to Lord et al., [3], regarding leadership, involves leadership perceptions existing in hierarchically organized cognitive categories or schema that can be represented by a set of prototypes. These prototypes or features are abstract concepts or ideas about shared features that are representative of a leader [17]. Individuals or followers use these features when rating leaders, i.e., followers match perceived behaviors or characters of the leader with the prototypes when rating their leaders [18]. Such an approach taps into the perceptual processes that underpin the leadership ratings or assessment at the expense of more concrete or measurable leadership effective and performance outcomes [19]. Therefore understanding the role and impact of these implicit prototypes on the leadership ratings provides an important avenue to understanding the rater and their ratings or assessment better.

#### Measuring methods

A range of questionnaires has been developed to measure the implicit theories of leadership. These include Lord et al.’s [3] 59-item scale, the Schein Descriptive Index [SDI: 20], the Campbell Leadership Indicator [CLI: 21]; ILTs scale [6], Leaders Described as Worthy of Influence [22], and Culturally Endorsed Implicit Leadership Theories [CILT: 23]. Epitropaki and Martin [5] further developed and tested the generalizability of Offermann et. al.,’s [6] 41 items scale of ILT which consisted of both core prototypic and anti-prototypic trait factors. Their study supported a valid 6-factor structure (i.e., sensitivity, intelligence, dedication, dynamism, tyranny, and masculinity) across several organizational setting in a shortened version of 21 items. This research used this 6-factor structure questionnaire. The use of questionnaires to measure the ILTs is moving towards an advanced stage, and this research will build on this work.

Both exogenous and endogenous thinking modes rely heavily on the eyes for information to process. After all, the eyes are the pre-eminent source of sensory information for the brain [24]. As a result, the movement of eyes offers the opportunity to glimpse into the cognition of individuals [25]. The eyes acquire more information than the brain can process. Selective visual attention enables the brain to extract relevant information in a quick and efficient manner and involves both conscious and subconscious processes [26]. Such an extracting process represents a fundamental premise of eye tracking research. What people look at, is what they are thinking of, or dealing with. According to Duchowski [27], eye tracking gives us insights into what is attracting the attention of an observer or what they are finding interesting. However, all such pieces of information are not treated the same [28] and partially integrated via the automatic-intuitive process of memory and perception [29, 30].

According to Hartmann and Fischer [31], eye-tracking or eye movement research has two simple assumptions that enable cognitive researchers to ‘read the mind.’ These are that our gaze indicates the object and the intensity of interest in that object [25, 32]. These assumptions have been extensively supported by reading research that links successively fixated and fixation duration to ease of comprehension [33]. Eye-tracking technology is very useful in investigating process model because they provide a wealth of dependent measures [28], which are objective and unobtrusive measure of what is being processed within a defined period [31]. Eye tracking research has been applied to a range of different subject matter, which includes reading, scene perception, usability testing, aviation, or driving [see 34]. All social species, such as humans, use eye gazing as a very powerful cue for others [35]. “An eye tracker can be a powerful tool that gives us a highly accurate representation and understanding of an individual’s eye movement behavior. Three attributes of *location*, *duration*, and *movement* form the basis for this understanding” [36]. The eye tracking provides a powerful tool. By measuring the eyes, the technology gives previously un-captured information, while at the same time is unobtrusive and often unconsciously done.

### Linking implicit leadership models to eye tracking

According to Perez et al. [37], eye movement analysis is optimal when coupled with additional tests, which can assess stimulus. How individuals respond to a stimulus can be recorded via questionnaire items, which can then be examined to provide evidence of cognitive or thinking processes [38]. Thus, combining eye tracking with established models of implicit leadership should provide a bridge and insights into cognition resulting from an individual’s gaze. The tracking of eyes and the implicit leadership model allows the assessment of the validity of constructs and provides evidence of the thinking processes involved in the response processes [38]. Previously Smith and Foti, [10], have suggested that ILT is most likely to operate in a context-sensitive, and is dynamic in nature, rather than static. Video can create assessable situations, which enable replicable and standardized experiences for a range of participants to assess. Subsequently, combining eye tracking, implicit leadership questionnaire, and a short video provide three elements within an experiment that can be either accurately measured or controlled. The movement eyes, ILT, and short videos are all recognized aspects of valid and reliable research and are effective methods for measuring key aspects of the leadership phenomena, which are sometimes simply ignored (e.g., the context).

The majority of leadership research simply assumes that what numerous followers are rating is approximately the same (i.e., their leaders) and ignores the physicality that individual experience during the process of leadership [39]. Research has neglected this variation and makes the assumption their experience is approximately similar, or context was too difficult to control. Video clips overcome this limitation and provide standardized and replicable visual imagery and sounds of leadership, and thus, move the measurement of leadership beyond its current over-reliance on retrospective perceptions of leaders and followers. In fact, Podsakoff, et al., [40] have strongly supported the use of videos in the testing and confirming the validity of constructs. An obvious strength of videos is their capacity to overcome the fleeting nature of most observations [41] and have been demonstrated to gain and maintain student attention and interest in organizational behavior lectures [e.g., 42]. The integration of video and eye tracking techniques has occurred in a range of studies, such as the ability of eye witnesses to identify the perpetrators of an observed crime [e.g., 43]. The current study is focused on establishing whether there is a significant measureable relationship between implicit leadership factors and facial features. Once this is established the development of experiential projects with leadership interventions can be implemented.

### How to examine the faces of human?

Video can easily represent the human face of leaders and followers. The human face is one of the most important types of visual stimuli in our environment, and its recognition is one of the most important cognitive tasks of daily human life [44]. The facial expression of humans commonly appears alongside other verbal and nonverbal cues (e.g., gaze, head orientation, gestures, and speech). “A key feature of facial behavior is its dynamic nature” which enables decoders (e.g., leaders and followers) to identify specific emotional states with greater coherence regarding intensity and arousal [45]. According to Xiao et al., [44], our experiences with faces in social interactions enables us to develop highly efficient ways process faces and identify characteristics such as age, sex, race, and identity itself. However, not all faces are treated the same. Several researchers have suggested that the role of facial cues in social judgments is greater than any other physical characteristics [46, 47]. Sell et. al., [48], suggest that humans have a built-in mechanism to recognize leadership suitability quickly and the face serves as a highly diagnostic tool to identify leaders in particular situations.

The Other Race Effect (ORE) suggests that how a face is scanned is dependent on race, and according to Nusseck et al., [49] may produce different emotional appraisals (i.e., happy and sad expression) from the same expression. Further, studies by Fu, Hu, Wang, Quinn, and Lee [50], Hu, Wang, Fu, Quinn, and Lee [51], and Hu, Wang, Han, Weare, and Fu [52] have all identified that Chinese adults fixated more on the eye regions of Caucasian faces than on eye regions of Chinese faces, while they fixated more on nose regions of Chinese faces than on that of Caucasian faces. The majority of facial recognition studies that have used videos suggest it occurs between 12 and 200 seconds [53–55]. In face recognition, differential racial experience leads to advantages and biases [56]. However, perceptual exposure to faces of other races, does not necessarily lead to a reduction in this other race effect [57]. Making eye contact allows for faster access to stored, categorical social information, such as gender and race [58].

### Leadership and cognition

According to Brown [59], leadership scholars are loosely bound together by a commonly held assumption, which Antonakis, Cianciolo, and Sternberg [60] describe leadership as the outcome of influence processes within a given context between two parties (i.e., leader and follower). Lord and Maher [14] have long suggested that this type of influencing process can be best understood from the social cognitive field perspective. A fundamental idea in this field is the dual process paradigm which represents two distinct information processing modes of cognition or thinking. An implicit or automatic mode that is based on simple associations and habitual responses stored in long-term memory, such as scripts, stereotypes, and heuristics. Such a stimulus-driven mode is involuntary, bottom-up thinking or control and is known as exogenous, but can also be referred to as system one type thinking.

The alternative mode is the goal-driven and involves conscious aware and the ability to access widely distributed, relevant information residing in different brain regions. Such goal-driven, voluntary top-down thinking or control is known as endogenous or system two type thinking. The endogenous mode has obvious advantages over the exogenous mode, regarding the quality of cognition. However, such capacity is reliant on the availability working memory and conscious awareness [61], which is slow, and requiring effort and motivation on the part of the social actors involved [59]. The system two type thinking can thus only occur once capacity is available.

### The research problem

The current study investigates the relationship between implicit leadership factors, as defined by Epitropaki and Martin [5] and the eye movement of follower observers. Implicit leadership factors are believed to be developed from an early age, as individuals become socialized by their parents, family, community, and society. A key aspect of this socialization is the learning about who is influential and their use of emotions. The expression of emotion is a powerful tool that enables social influence [62] [63], which enables leaders and followers to both communicate and distil information about their feelings, attitudes, and intentions [64] [65] [66]. In fact, to be successful in functioning within most social interactions (e.g., leadership) requires the appropriate processing of emotions [67] and individuals rapidly make attributions about emotions from the face [68]. Rule and his colleagues [69] [70] have suggested that the perceived face of leaders alone may account for leadership success. Recently, Schurgin et al., [71] identified five facial features which accounted for almost 90% of all eye fixations relating to emotions, which like implicit leadership, such behaviors are likely to have developed from an early age through socialization.

Hypothesis One The implicit leadership prototypes of sensitivity, intelligence, dedication, and dynamism should have a direct relationship with the facial features, such as the left eye, right eye, nasion, upper nose, lower nose, and upper lip

Dominant type behaviors such as tyranny and masculinity have had a long recognized association with leadership and facial appearances [72] and are classified within the implicit leadership model as anti-prototypes. Dominant facial features may signal physical leadership potential [73]. The lower nose is a dominant facial feature and is likely to be associated with tyrant and masculinity.

Hypothesis Two The implicit leadership anti-prototypes of tyrant and masculinity should have a direct relationship with the facial feature of the lower nose.

## Method

### Ethics

This study was conducted in Malaysia according to the Monash University Human Research Ethic Committee (MUHREC) principles and guidelines of Monash University and was approved as project CF15/4671–2015002014. Participants were first provided with an explanatory statement that enabled them to give informed consent, in terms of knowing what the research involves, why they were chosen, what consent means, possible benefits and risks, and assured confidentiality. Participants then signed a consent form approved by the MUHREC committee. Each participant was given a voucher worth 15 Malaysian ringgits for their involvement.

### Participants

The convenience sample of 81 Asian participants was drawn from an Australian University embedded in the South East Asia region. The majority of participants were Chinese (62%) with Indian, Malaysian and Indonesian making up the remaining 38%. Females accounted for 69%, and the average age was 28. The sample can be further described in terms of local undergraduate students (32%), local postgraduate students (14%), international undergraduate students (11%), international postgraduate students (8%), university professional staff (14%), and academic staff (22%).

### Materials

The current study used a Tobii 1750 eye tracker (0.5-degree precision, 17 inches, 50 Hz sample rate, and 1280x 1024 pixels resolution) to record participants’ fixation on a 30-second video of a suggested leader. The Tobii Studio Program was used to control the stimulus presentation and recording of the questionnaire and demographics. We used Epitropaki and Martin [5], six factors, 21 items, Implicit Leadership Theory Questionnaire, which measures both leadership prototypes and leadership anti-prototypes. Participants were asked to assess how characteristic were these items in terms of a leader on a seven-point Likert scale (i.e., 1 –“not at all characteristic,” 4 –“neutral,” and 7 –“extremely characteristic”). The shortened version of the ILT has obvious advantages, such as less items which reduces the effort and time required to complete. Epitropaki and Martin’s [5] shorten version has a confirmed factor structure, which is generalizable and remained stable over time. The factors and their items for these leadership prototypes were (a) sensitivity–(3 items: sincere, helpful, understanding); (b) intelligence–(4 items: intelligent, educated, clever, and knowledgeable); (c) dedication–(3 items: dedicated, motivated, and hardworking); and (d) dynamism–(3 items: energetic, strong, and dynamic). The factors and items for these leadership anti-prototypes were (a) tyranny–(6 items: domineering, pushy, manipulative, loud, conceited, and selfish); and masculinity–(2 items: male and masculine).

We used a 30-second video of a seated Caucasian female, whose head, upper body, and arms/hands were clearly visible, as she detailed and read out aloud her career highlights (i.e., her philosophy and successes). She did not actively engage with the camera, and thus the participant observed no direct eye contract. Making eye contact, enables individuals to access faster stored, categorical social information about the gender and race [58]. Also, the lack of any direct gaze inhibits the modulation of subsequent brain activation and cognitive processes [74]. As a result, such non-display of leadership or influence behaviors by our female presenter should inhibit the activation of System Two Type Thinking. However, the video was dynamic in nature, which enabled facial expressions and verbal and nonverbal cues (i.e., gaze, head orientation, gestures, and speech) to be consistently observed.

### Procedure

The experiments took place in a quiet room with consistent illumination for each experiment. Each participant completed the study individually and was first surveyed to ensure their eyes were capable of adequately seeing the screen [see 75]. If successful, the participant was then seated comfortably at a desk, 64 cm from the eye tracker screen, which had a mouse connected to the computer running the Tobii study program. The eye movements of the participant were then calibrated in according to the recommendations of Tobii, and if no error vectors were present, the participant was invited to begin, via clicking the mouse. This resulted in the participants being first invited to complete Epitropaki and Martin’s [5] Implicit Leadership Theory Questionnaire, via 21 questions that individually appeared on the screen, which were then answered via a mouse. Upon completing these questions, the participant was informed that they would now view a 30-second video of a leader, and upon clicking the mouse, the video was displayed. After participants completed watching the video they completed a demographics survey.

The recorded eye movements of participants were analyzed using Tobii Studio Program, which involved identifying five facial regions or areas of interest or AOI (Eyes, Nasion, Upper and lower Nose and Upper Lip). In according with Schurgin et al., [71], findings that the eyes, upper nose, lower nose, upper lip, and nasion (i.e., area between the eyes) accounted for 88.03% of all fixations, particularly when individuals were seeking out different emotional cues within a face. This finding is consistent with Yabus [76], seminal finding that a typical face search pattern is triangular in nature and involve both eyes, nose, and mouth. The AOIs were altered in each frame to ensure accuracy and no overlapping (see S1 Fig). The results from the frequencies of AOI fixations and the on-line questionnaire were analyzed using SPSS to determine relationships.

## Results

The ILT questionnaire factors and their items for these leadership prototypes were (a) sensitivity–(3 items: sincere, helpful, understanding), which had a Cronbach Alpha of .69; (b) intelligence–(4 items: intelligent, educated, clever, and knowledgeable) which had a Cronbach Alpha of .70; (c) dedication–(3 items: dedicated, motivated, and hardworking), which had a Cronbach Alpha of .63; and (d) dynamism–(3 items: energetic, strong, and dynamic), which had a Cronbach Alpha of .54. The factors and items for these leadership anti-prototypes were (a) tyranny–(6 items: domineering, pushy, manipulative, loud, conceited, and selfish), which had a Cronbach Alpha of .73; and masculinity–(2 items: male and masculine), which had a Cronbach Alpha of .76. These factors will thus be correlated with the AOI of the face to assess if there is a correlation.

To examine the participants’ implicit models of leadership and their frequency of fixations on the AOIs, we conducted a Pearson correlation analysis. Table 1 reveals significant correlations of between r -.28, p < .05 and r .57, p < .01 for all prototypes and anti-prototypes implicit leadership factors, except for masculinity with sensitivity, intelligence, dedication, and dynamism, and tyranny with intelligence and dedication. Such findings are consistent with previous studies [see 5].

**Table 1:** Correlations of implicit leadership theory factors and frequency of visits to areas of interest (n = 81).

	1 Sensitivity	2 Intelligence	3 Dedication	4 Dynamism	5 Tyranny	6 Masculinity
**Prototypes**						
**1. Sensitivity**	1.00	.35**	.55**	.39**	-.28*	-.24
**2. Intelligence**	.35**	1.00	.57**	.54**	.21	.14
**3. Dedication**	.55**	.57**	1.00	.50**	.02	-.13
**4. Dynamism**	.39**	.54**	.50**	1.00	.27	.11
**Anti-Prototypes**						
**5. Tyranny**	-.28*	.21	.02	.27*	1.00	.35*
**6. Masculinity**	-.24	.14	-.13	.11	.35*	1.00
**Areas of interest: Face**						
**7. Left Eye**	.04	.11	.18	-.12	-.14	.04
**8. Right Eye**	.12	-.10	.00	.04	-.20	-.17
**9. Nasion**	.30*	.01	.11	.08	-.30*	.05
**10. Upper Nose**	.28*	.09	.16	.18	-.24	.21
**11. Lower Nose**	.20	.20	.24	.18	-.07	.28*
**12. Upper Lip**	.01	.36*	.29*	.20	.17	.06
**13. None**	.09	.03	-.11	.01	-.29*	.03

Note

*. P < .05 (2-tailed)

** = P<. 0.01 level (2-tailed); Likert scale = 1 = Not at all characteristic, 4 = Neutral, and 7 = extremely characteristic

Four implicit leadership prototypes were correlated against the AOIs. The prototype sensitivity was positively and significantly correlated with two AOIs, which were nasion (r = .30, p < .05) and the upper nose (r = .28, p < .05). In other words, how characteristic participants view the prototype of sensitivity for leadership, the more likely the nasion and upper nose features of the face will be focused on. Such findings support previous research that suggests that Chinese adults fixated more on the eye regions of Caucasian faces [50–52]. Interestingly, no correlations between either the left or right eye AOIs with any of the prototypes and anti-prototypes were identified.

The prototype of intelligence (r = .36, p < .05) and dedication (r = .29, p < .05), were positively and significantly correlated with the upper lip. In other words, how characteristic participants view the prototypes of intelligence and dedication for leadership, the more likely, their fixations on the upper lip feature of the face will occur. The lack of direct eye contract presented in the video may account for the no correlations between the AOI of either eye.

Two anti-prototype of leadership were correlated against AOIs. The anti-prototype of tyranny was negatively and significantly correlated with nasion (r = -.30, p < .05). In other words, how characteristic participants’ view the anti-prototypes of tyranny for leadership, the less likely, their fixations on nasion facial feature will occur. The anti-prototype of masculinity was significantly correlated with the lower nose (r = .28, p < .05). In other words, how characteristic participants view the anti-prototype of masculinity for leadership, the more likely, their fixations on the lower nose facial feature will occur.

The nasion feature had a bi-directional relationship with the prototype of sensitivity (r = .30, p < .05) and the anti-prototype of Tyranny (r = -.30, P < .05). This bi-directional relationship highlights the level of importance the participants place on sensitivity versus tyranny. Therefore, revealing the participant potential bias toward a particular implicit leadership behavior.

## Discussion

The results of the present study support our hypothesis that different implicit leadership factors have significant relationships with various facial features when participants view a reputed leader. Such findings support the assertion that implicit leadership theoretical factors influence more than just an individual’s perceptional and attitudinal rating of leaders, but physically[83], i.e., their gaze scanning patterns. As previously highlighted, the implicit leadership prototypes / anti-prototypes and eye movement behaviors develop very early in one’s life through a process of socialization involving, being influenced and influencing others. These types of implicit leadership appear to have a relationship that helps direct the attention of followers, and may involves conscious and subconscious processes [26]. Examining such extracting processes provides important insights into what is attracting attention, but some caution needs to be observed, since automatic-intuitive memory and perceptional processes are involved [see 29, 30].

The use of a non-engaging, reputed leader within the current study, via the video, helps provide a deeper understanding of how mental representations like ILT operate. By suggesting to participants that they are going to view a leader, we are encouraging them to subordinate themselves to this leader, which according to Brown [59], is a quintessence aspect of leadership. Such subordination to others is more likely to emphasize system one type thinking (i.e., automatic and unconscious cognitive processes). According to Smith and DeCoster [77], the connective architecture of system one typically operates in a schematic knowledge framework which focuses on the ‘here’ and ‘now,' rather than anticipating future consequences [78]. The selection of a non-typically leader who does not engage with the rater means that leadership schemas or expectations are less likely to be activated. This encourages bottom-up or stiumulus driven cognitions of simple associations and habitual responses (i.e., system one type thinking). The inclusion of leadership behaviors, such as ‘visioning’ would more likely encourage the participant to use their system two type thinking. Thus, the current study has attempted to isolate and focus on system one type thinking, to more clearly understand how the relationships between ILT and gaze scanning. Overall, the study findings suggest that leaders who do not directly engage with them (i.e., not using leadership or influential behaviors, such as eye contact) are more likely to be rated according to the ILT. In other words, the observers may be rating their leaders based on simple associations and habitual responses, which may not adequately represent the leadership potential or capacity of the leader being observed. As this capacity is not demonstrated, then they apply their ILT to the situation to try and develop context.

“An individual’s gaze is a key tool in initiating, sustaining and ending social interactions” [79]. Such a tool enables the intention of others, regarding collaboration, coordination, social learning, dominance, and leadership to be understood [35, 80, 81]. Unfortunately, the gaze provides more information than the brain can process and thus, relies on ‘selective visual attention’ to cope [26]. As a result, individuals are looking for gaze signals from the eye region to understand a person’s focus of attention, mental state, and intentions [82]. Thus, the findings of two positive and significant relationships between prototype of sensitivity, and (a) the nasion, and (b) the upper nose regions are not surprising. In simple terms, the prototype of sensitivity in leaders may be influenced by the direction of their head as indicated by their nasion and upper nose. After all, the particular direction that an individual looks, is an indication of attention and influence [83]. Perhaps the observers are seeking to determine how sensitive the leader is to them, i.e., via how much they are looking in their direction. A similar relationship between the anti-prototype of tyranny and the nasion may also exist. For example, the less a leader looks in your direction, the more likely you are going to have the impression of being unconnected with them, which may lead to activating scripts relating to isolation or being outside the group and consequently, seeing them as tyrants. The current study also found that the leadership anti-prototype of tyranny was negatively and significantly correlated with the nasion face feature, which is the opposite to the relationship of sensitivity to the nasion feature.

Emotional expression plays a significant role in any successful social interactions, which according to Schyns, Petro, and Smith [84], may explain why individuals have a remarkable ability for rapidly and efficiently decoding of them. Emotions have long been recognized as an importance influence in the leadership process [85], and has generated a stream of research investigating the relationship between emotional intelligence and leadership. Schurgin et al., [71] have previously highlighted the importance of the upper lip and other facial features in identifying the emotions of joy, disgust, fear, anger, sadness, and shame. The finding of the current study that the prototype of intelligence and dedication had positive and significant relationships with the upper lip appears to suggest that participants are making inferences based on the upper lip. The examinations of the inferences that followers make from observing the emotions of their leaders are rare, and none have been found that directly observe the upper lip. However, recently Caza, Zhang, Wang, and Bai, [86] identified that followers make judgments about leaders’ trustworthiness and emotional sincerity from the observation of their emotions. In terms of trustworthiness, followers may be attempting to determine the level of ability and competence of their leader [87] through the observation of emotions [88]. While in terms of emotional sincerity, followers could be attempting to determine how genuine are the emotions being expressed by their leaders [72] In other words, since followers are unable to directly assess the internal states of their leaders, they make judgments based on their assessment of emotional sincerity.

The current study found that the leadership anti-prototype of masculinity was positively and significantly correlated with the lower nose. This finding supports the link between masculinity and facial appearance [68]. The importance of dominances to leadership has long been recognized. From a human evolutionary perspective, the facial cue of dominance may signal leadership potential, in terms of physical features that match leadership prototypes and anti-prototypes of followers [72]. Therefore, the link between the lower nose and masculinity may suggest some evolutionary need of humans, which has linked the lower nose with the need to identify dominance for survival or adaptation.

## Conclusion

The use of a non-engaging, reputed leader within the current study, via the video, helps provide a deeper understanding of how mental representations like ILT operate. By suggesting to participants that they are going to view a leader, we are encouraging them to subordinate themselves to this leader, which according to Brown [59], is a quintessence aspect of leadership. Such subordination to others is more likely to emphasize system one type thinking (i.e., automatic and unconscious cognitive processes). Unfortunately, we are unable to truly control or identify which of the dual thinking systems the participants is actually using.

A number of limitations of this study exist, such as only using two methods of measurement, i.e., eye tracking and questionnaire. However, the study does add to the literature in terms of Other Race Effect and female leaders along with identifying the fixation relationship between key facial features and prototypes and anti-prototypes of implicit leadership. Future research needs to investigate the relationship of stimulating and engaging leaders to the implicit leadership factors and movement of followers’ eyes. After all, our gaze is a key tool in initiating, sustaining and ending social interactions [79]. We hope the findings of this study encourage others to investigate further the relationship between implicit leadership models and the cognitive processes influence the rating of others.

## Supporting information

S1 Fig A single frame from the 30-second video with the Areas of Interests (AOIs) illustrated on the face. (TIF)
