# Supplementary figures and images for: Does the implicit models of leadership influence the scanning of other-race faces in adults?
Source: PLoS One. 2017 Jul 7;12(7):e0179058. doi: 10.1371/journal.pone.0179058 (PMC5501397; doi:10.1371/journal.pone.0179058)

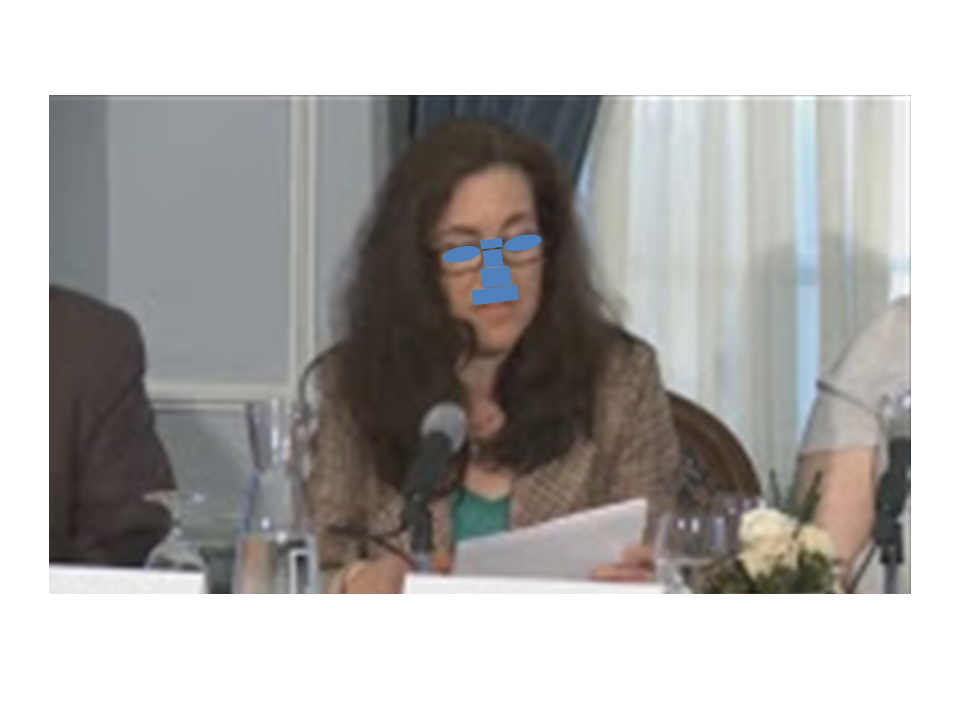

Supplement: S1 Fig — (TIF) [file pone.0179058.s001.tif]
